# Genetic evidence for common pathways in human age-related diseases

**DOI:** 10.1111/acel.12362

**Published:** 2015-06-15

**Authors:** Simon C Johnson, Xiao Dong, Jan Vijg, Yousin Suh

**Affiliations:** 1 Department of Genetics, Albert Einstein College of MedicineBronx, NY, USA; 2 Department of Ophthalmology and Visual Sciences, Albert Einstein College of MedicineBronx, NY, USA; 3 Department of Medicine Endocrinology, Albert Einstein College of MedicineBronx, NY, USA

**Keywords:** ageing, aging, genetics, gerontogenes, human, inflammation, Ink4a, longevity gene

## Abstract

Aging is the single largest risk factor for chronic disease. Studies in model organisms have identified conserved pathways that modulate aging rate and the onset and progression of multiple age-related diseases, suggesting that common pathways of aging may influence age-related diseases in humans as well. To determine whether there is genetic evidence supporting the notion of common pathways underlying age-related diseases, we analyzed the genes and pathways found to be associated with five major categories of age-related disease using a total of 410 genomewide association studies (GWAS). While only a small number of genes are shared among all five disease categories, those found in at least three of the five major age-related disease categories are highly enriched for apoliprotein metabolism genes. We found that a more substantial number of gene ontology (GO) terms are shared among the 5 age-related disease categories and shared GO terms include canonical aging pathways identified in model organisms, such as nutrient-sensing signaling, translation, proteostasis, stress responses, and genome maintenance. Taking advantage of the vast amount of genetic data from the GWAS, our findings provide the first direct evidence that conserved pathways of aging simultaneously influence multiple age-related diseases in humans as has been demonstrated in model organisms.

## Introduction

**Fig 1:**
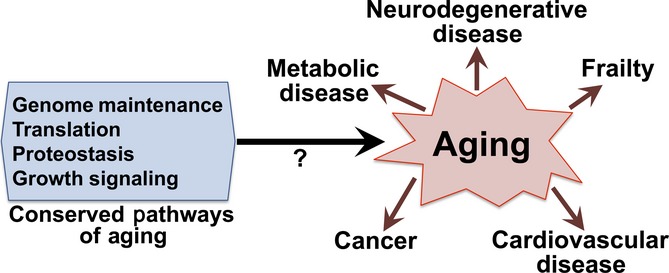
Common pathways in aging and age-related disease. Aging is the single greatest risk factor for age-related disease, and it is well established that conserved genetic pathways of aging impact multiple age-related pathologies in model organisms, but the role of conserved pathways of aging in human age-related disease is unclear.

Aging is a complex process of progressive functional decline influenced by environmental, genetic, and stochastic factors (Lopez-Otin *et al*., 2013). Genetic approaches to studying aging using yeast, nematode, fly, and rodent models have identified conserved genetic factors that modulate aging (Kenyon, 2010; Vijg & Suh, 2013). These include genome and epigenome maintenance; nutrient-sensing signaling through insulin/IGF-1, mTOR, and AMP kinase; regulation of proteostasis through cellular degradation pathways and activity of the unfolded protein responses; inflammation and senescence pathways; and key transcriptional regulators such as the Foxo transcription factors. In model organisms, alterations to these conserved genetic modifiers of aging can impact both survival (lifespan) and health span, the percentage of life spent free from significant pathology. Alterations to genome maintenance, inflammation, or proteostasis can result in shortened lifespan and symptoms of rapid aging (Merkwirth *et al*., 2012; Schleit *et al*., 2013; Jurk *et al*., 2014), while genetic, dietary, and pharmacological interventions that reduce growth signaling through IIS, mTOR, and AMPK tend to increase lifespan and delay and reduce the frequency and severity of aging-related pathologies. Thus, interventions designed to target the underlying mechanisms of aging are expected to provide great benefit to human health by attenuating a broad range of pathologies (Fontana *et al*., 2010; Berry & Cirulli, 2013; Johnson *et al*., 2013; Torgovnick *et al*., 2013; Sikora, 2014). While the notion that age-related diseases are driven by common underlying pathways of aging is supported by model organism studies (Fig.1), whether it remains true in human aging is unclear.

Genomewide association studies (GWAS) have provided unprecedented opportunities to identify genes and genomic regions associated with complex traits in human populations, including disease risk. At the time of this study, 1738 GWAS had been conducted for 816 human traits (including human diseases), together reporting 11 533 trait-associated single-nucleotide polymorphisms (SNPs) reaching a suggestive association threshold of *P* < 1 × 10^−5^ (see the Web resource of GWAS catalog: http://www.genome.gov/gwastudies). These results have greatly contributed to our understanding of biological pathways involved in individual human traits. In contrast, little insights have been gained from GWAS regarding the genetic pathways regulating human longevity. To date, 5 GWAS of longevity, involving long-lived individuals compared to control populations, have been reported in the catalog of published GWAS. Among these studies, the APOE gene has emerged as the only consistently replicated locus associated with human lifespan. Human health span has not been studied by GWAS, and human age-related diseases have been studied only as independent traits. Here, we have taken a novel approach to jointly analyzing a large set of GWAS data to directly address the question of whether human age-related diseases are linked by common underlying pathways of aging as has been demonstrated in model organisms.

## Results

To approach the question of whether age-related diseases are influenced by common genetic pathways, we downloaded all available GWAS data from the complete (November 9, 2013) release of the NHGRI GWAS database (Welter *et al*., 2014). To focus our analysis on traits that have been sufficiently studied, as well as to address computational limitations in downstream analyses, we chose to restrict our analyses to traits that have been examined in a minimum of five independent GWAS, resulting in a total of 410 GWAS in the 5 age-related disease categories. Furthermore, in each GWAS, we only considered SNPs with *P*-value less than 1 × 10^−5^ as trait associated. The combination of these two criteria ensures that only significant SNPs from well-studied traits are represented in the work presented here. We defined each trait meeting these criteria as either age-associated (Table1) or non-age-associated (Table S1, Supporting information). Age-associated traits were then assigned to one of five broad and well-established age-related disease categories: cardiovascular disease, metabolic disease, neurodegenerative disease, cancer, and collectively other age-related traits [see (Perez-Lopez *et al*., 2009; Walter *et al*., 2011; Martin, 2012; Johnson *et al*., 2013; Brunet & Berger, 2014)]. The other age-related trait category includes traits conventionally associated with aging, such as telomere length and inflammation, as well as related age-associated diseases including rheumatoid arthritis and age-related macular degeneration. GWAS traits, properties, and assignments are indicated in Table1 (age-associated traits).

**Table 1:** Genomewide association studies diseases used in this study

Disease	Number of GWAS	Significant SNPs	Disease category
Adiponectin levels	9	44	Metabolic
Age-related macular degeneration	9	66	Other
Alzheimer’s disease	18	64	Neurodegenerative
Alzheimer’s disease (late onset)	9	35	Neurodegenerative
Amyotrophic lateral sclerosis	12	54	Neurodegenerative
Atrial fibrillation	5	20	Cardiovascular
Blood pressure	5	57	Cardiovascular
Body mass index	17	124	Other
Bone mineral density	12	97	Other
Breast cancer	26	122	Cancer
C-reactive protein	8	46	Other
Colorectal cancer	14	47	Cancer
Coronary heart disease	17	133	Cardiovascular
Endometriosis	5	26	Cancer
Fasting plasma glucose	8	11	Metabolic
Glaucoma (primary open-angle)	5	12	Other
HDL cholesterol	12	100	Metabolic
Hypertension	8	33	Cardiovascular
LDL cholesterol	12	76	Metabolic
*Longevity*	*5*	*43*	^*^ (longevity)
Lung cancer	10	24	Cancer
Mean platelet volume	5	51	Other
Melanoma	7	18	Cancer
Metabolite levels	8	98	Metabolic
Multiple sclerosis	16	162	Neurodegenerative
Myopia (pathological)	7	76	Other
Obesity	7	77	Metabolic
Osteoarthritis	5	8	Other
Pancreatic cancer	5	39	Cancer
Parkinson’s disease	13	67	Neurodegenerative
Prostate cancer	19	104	Cancer
Pulmonary function	5	55	Cardiovascular
QT interval	8	76	Cardiovascular
Rheumatoid arthritis	14	78	Other
Telomere length	7	16	Other
Triglycerides	12	79	Metabolic
Type 2 diabetes	32	141	Metabolic
Urate levels	9	68	Metabolic
Uric acid levels	5	34	Metabolic

Diseases/traits that met the criteria for inclusion in this study are indicated with the number of GWAS studies, number of GWAS significant SNPs, and disease category assignment as indicated. The trait longevity, while meeting our criteria for inclusion, was not included.

### A gene-based assessment of age-related diseases

**Fig 2:**
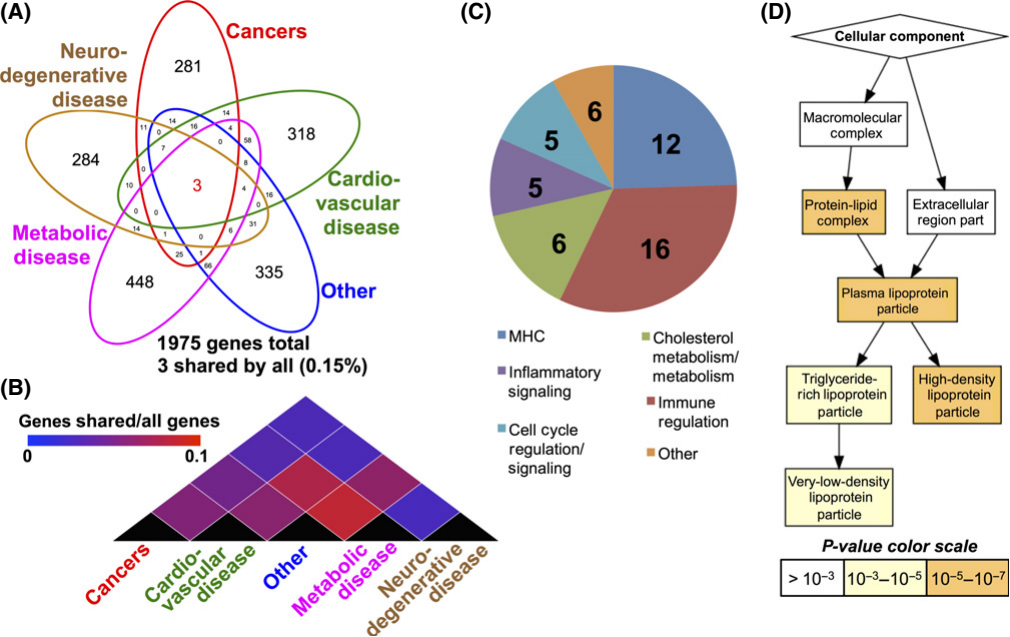
Genomewide association studies (GWAS) significant genes shared among age-associated diseases. Age-related disease categories show very little overlap of GWAS significant genes (A), with only 3 (0.15%) of genes identified shared among all groups, while the majority of genes appear to be detected in only one group. (B) Clustering of age-related disease categories by similarity shows that cardiovascular disease, metabolic disease, and other age-related pathologies are closely related, while cancers and neurodegenerative diseases represent the most distinct groups. (C) Number and category of genes shared among at least 3 age-related disease categories represent those predominately involved in immunity, inflammation, cell cycle regulation, and cholesterol metabolism. (D) GO analysis of these genes compared to the background set of all 1975 GWAS detected genes revealed that apolipoprotein metabolism is significantly and specifically enriched in this gene set.

GWAS SNPs were assigned to genes (see Methods) resulting in a total number of 1975 unique genes among all five categories (Fig.2). Only three genes (0.15% of total genes) were shared among all the five age-related diseases categories (Fig.2A, Table2). All three shared genes fell in the MHC locus, a highly variable region over-represented in GWAS studies. Clustering of age-related disease groups by gene list similarity (fraction of genes shared vs. total) indicates that cardiovascular disease, metabolic disease, and other age-related trait categories cluster more closely, while cancer and neurodegenerative diseases represent the most distinct group with the least overlap between the two (Fig.2B). Metabolic disease and other age-related diseases show the highest similarity between two groups at an overlap of 0.083 (fraction of all genes), whereas metabolic disease and neurodegenerative disease show the least overlap at 0.029 (fraction of total genes).

**Table 2:** Genomewide association studies associated genes shared among 3 or more age-related disease categories

Gene ID	Gene name	Function
3118	Major histocompatibility complex, class II, DQ alpha 2	MHC
3119*	Major histocompatibility complex, class II, DQ beta 1	MHC
3120	Major histocompatibility complex, class II, DQ beta 2	MHC
3122*	Major histocompatibility complex, class II, DR alpha	MHC
3123	Major histocompatibility complex, class II, DR beta 4	MHC
100507714	HLA class II histocompatibility antigen, DQ beta 1 chain-like	MHC
100507709	HLA class II histocompatibility antigen, DRB1-7 beta chain-like	MHC
101060835*	HLA class II histocompatibility antigen, DQ beta 1 chain-like	MHC
100509457	HLA class II histocompatibility antigen, DQ alpha 1 chain-like	MHC
3117	Major histocompatibility complex, class II, DQ alpha 1	MHC
56244	Butyrophilin-like 2 (MHC class II associated)	MHC
84166	NLR family, CARD domain containing 5	MHC Regulation
7940	Leukocyte specific transcript 1	Immune Regulation
80740	Lymphocyte antigen 6 complex, locus G6C	Immune Regulation
80741	Lymphocyte antigen 6 complex, locus G5C	Immune Regulation
1379	Complement component (3b/4b) receptor 1-like	Immune Regulation
1460	Lymphocyte antigen 6 complex, locus G5B	Immune Regulation
199	Allograft inflammatory factor 1	Immune Regulation
259197	Natural cytotoxicity triggering receptor 3	Immune Regulation
259215	Lymphocyte antigen 6 complex, locus G6F;	Immune Regulation
5819	Poliovirus receptor-related 2	Immune Regulation
58496	Lymphocyte antigen 6 complex, locus G5B	Immune Regulation
58530	Lymphocyte antigen 6 complex, locus G6F	Immune Regulation
6934	Transcription factor 7-like 2 (T-cell specific)	Immune Regulation
10665	Erythroid Differentiation-Related Factor 1	Immune Regulation
28	ABO blood group	Immune Regulation
5089	Pre-B-cell leukemia homeobox 2	Immune Regulation
63940	G-protein signaling modulator 3	Immune Regulation
177	Advanced glycosylation end product specific receptor	Inflammation
4050	Lymphotoxin beta (TNF superfamily, member 3)	Inflammation
10019	SH2B adaptor protein 3	Inflammation
10554	Lysophosphatidic acid acyltransferase, alpha	Inflammation/Lipid Signaling
64116	Solute carrier family 39 (zinc transporter), member 8	Other
1029†	Cyclin-dependent kinase inhibitor 2A (p16)	Cell Cycle Regulation (p16 *INK4a*)
1030	Cyclin-dependent kinase inhibitor 2B (p15)	Cell Cycle Regulation
2262	Glypican 5	Cell Signaling
4855	Notch homolog 4	Cell Signaling
6311	Ataxin 2	Cell Signaling/Intracellular Trafficking
57827	Apoliprotein M	Cholesterol Metabolism/Cell Signaling
60526	Apolipoprotein B	Cholesterol Metabolism/Other
1071	Cholesteryl ester transfer protein, plasma	Cholesterol Metabolism
341	Apolipoprotein C-I	Cholesterol Metabolism
348	Apolipoprotein E	Cholesterol Metabolism
55937	Apolipoprotein M	Cholesterol Metabolism
217	Aldehyde dehydrogenase 2 family	Metabolism
3990	Lipase, hepatic	Metabolism
6048	Ring finger protein 5	Histone modification
6838	Surfeit 6	Nucleolar Protein/Ribosome Biogenesis
79068	Fat mass and obesity associated (FTO)	mRNA Regulation
10452†	TOM40	Mitochondrial Protein Import (APOE LOCUS)

Gene ID, name, and generic function, based on NCBI gene description, are indicated.

* —Genes associated with all five disease categories.

† —Genes previously reported to associate with multiple age-related diseases.

The low number of shared genes among all age-related disease groups is likely in part a result of the stringent statistical cut-offs of GWAS. To broaden our analysis to include genes that are shared in at least the majority of age-related disease categories, we assessed the genes shared by at least three of the five age-related categories resulting in a total of 50 genes (2.5% of unique genes, listed in Table2), including 12 MHC genes as well as a number of genes involved in inflammation, cell cycle regulation, and cholesterol/apolipoprotein metabolism (Fig.2C, Table2). Notably, both TOMM40/APOE, the highly reproducible longevity-associated locus, and p16INK4a, a cell cycle/senescence regulator gene locus previously identified to be associated with multiple diseases (Jeck *et al*., 2012), are represented in this gene set. A Gene Ontology (GO) analysis of these 50 genes against the baseline set of the total 1975 unique genes revealed a significant enrichment of GO terms in the apolipoprotein metabolism pathway (Fig.2D, Table S2, Supporting information). Strikingly, apolipoprotein metabolism is the only biological process significantly enriched in the gene-based overlap between the age-related disease categories defined in this work.

### A pathway approach to age-related disease

**Fig 3:**
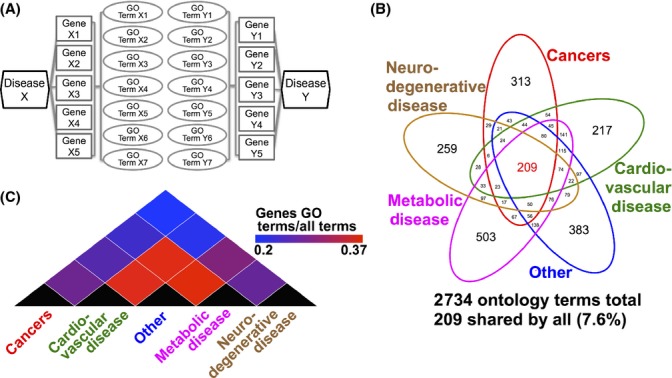
A pathway-based analysis of age-related diseases. (A) Age-related disease groups were analyzed using a pathway-based approach by examining the GO terms associated with genomewide association studies identified genes rather than the genes themselves. (B) A comparison between age-related disease categories shows a more significant similarity between ontology terms than was observed using individual genes (7.6% vs. 0.15%). (C) Although the relative percentage of overlapping terms is greater the relative similarities between disease categories are unchanged as determined by unsupervised clustering.

To test whether age-related diseases share common underlying genetic mechanisms and pathways, we next utilized a novel approach of assessing the pathway-based overlap between disease categories. For this purpose, we compiled GO terms for each identified gene in each group and assessed the overlap of GO terms between disease groups (Fig.3A). Assignment of GO terms resulted in a total list of 2734 unique GO terms among all 5 age-related disease genes (Fig.3B). Of these, 209 GO terms (7.6% of total) were shared among all five groups. Clustering of disease groups by similarity resulted in a pattern identical to that observed using gene-based clustering, indicating that the use of GO terms did not alter the qualitative comparisons between diseases. As with a gene-based approach, we found cardiovascular disease and metabolic disease are more closely related while cancer and neurodegenerative disease are the most distantly related (Fig.3C). Among the 7.6% of GO terms overlapped among all five age-related disease categories, there is a significantly higher than random similarity between terms based on the background (*P* < 1 × 10^−15^, one-sided Wilcoxon rank-sum test, see Methods for more details). Among the overlapping GO terms, we found clusters of terms related to nutrient-sensing signaling, translation, genome maintenance, proteostasis, oxidative stress responses, inflammation, and, as in the gene-based assessment, lipoprotein metabolism, and most of them are known to be canonical pathways of aging (Tables S3 and S4, Supporting information, Fig.4). A principle component analysis of these shared GO terms using REViGO term clustering and analysis further indicated the presence of shared canonical aging pathways in the 5 age-related disease categories (Fig. S2, Supporting information). Considering the stringent criteria for inclusion of individual GWAS traits and the use of only genes with significant trait-associated SNPs, the significant enrichment of canonical pathways of aging among the pathways shared in all 5 age-related disease categories is striking.

**Fig 4:**
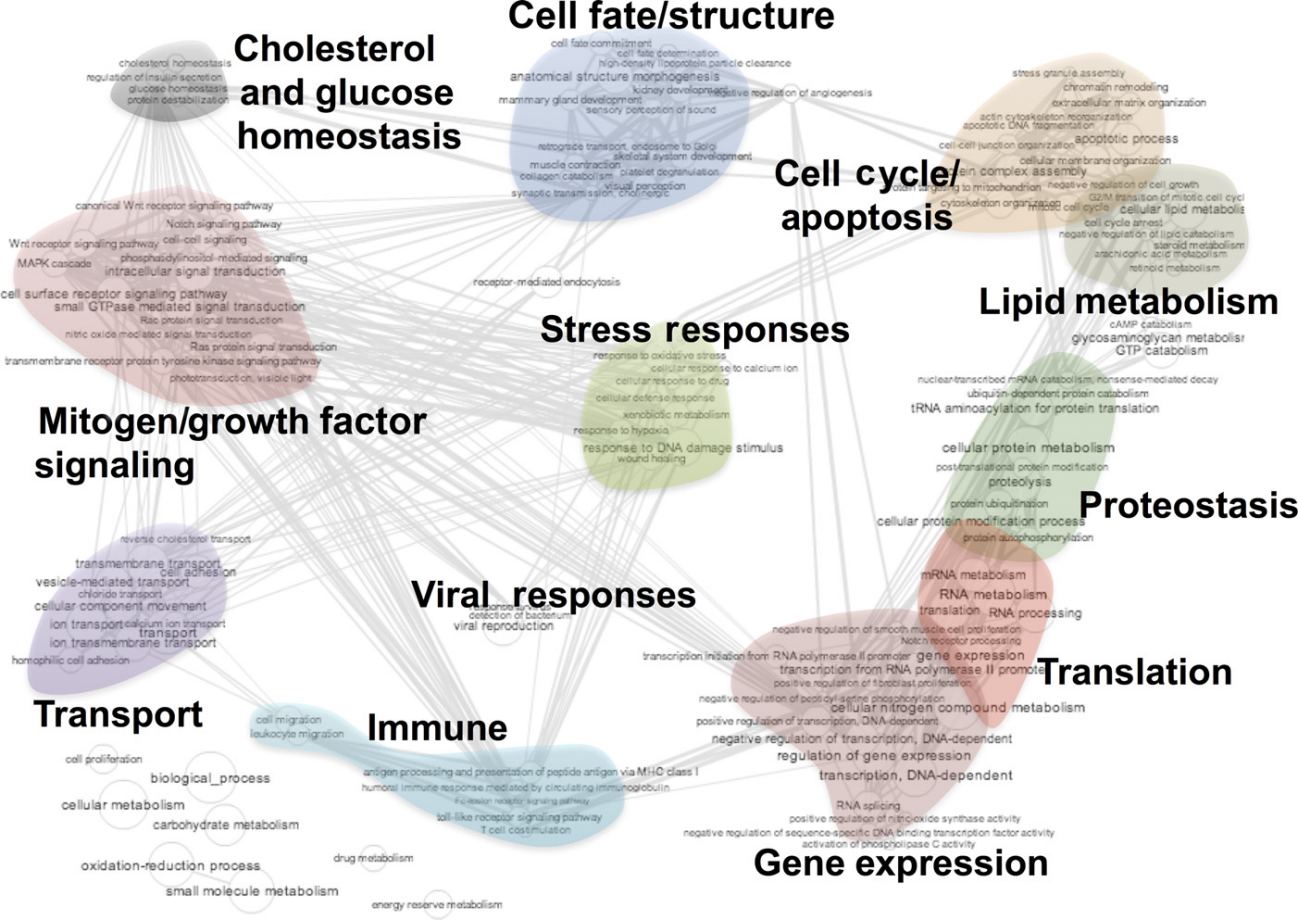
Pathway analysis identified shared pathways among age-related diseases. Visualization of the GO terms shared by all five age-related disease categories reveals common pathways in human age-related disease. These include many canonical aging pathways identified in model organisms, such as nutrient-sensing signaling, proteostasis, and stress responses, as well as cholesterol metabolism, as was identified in the gene-based analysis. GO terms shared among age-related diseases are significantly enriched for similar terms compared to the background (*P*-value < 10^−15^, one-sided Wilcoxon rank-sum test, see Methods).

For reference, we examined the genes associated with the GWAS trait longevity at *P* < 1 × 10^−5^ and the GO terms associated with this gene set (Tables S5 and S6, Supporting information). Lipoprotein metabolism pathways reach nominal significance for enrichment among this gene set, although no GO term is significant after multiple testing correction.

### Statistical assessment

Our approach of identifying genes associated with a significance level of *P* < 1 × 10^−5^ in multiple independent GWAS traits is amenable to the Fisher’s combined probability test (Li *et al*., 2014). This test indicates that the combined *P*-value for 3 independent tests with a cut-off of *P* < 1 × 10^−5^ each is equal to 6.32 × 10^−13^, while the combined *P*-value for 5 independent tests each with a cut-off of *P* < 1 × 10^−5^ is 4.9 × 10^−20^. Furthermore, a comparison of genes or GO terms identified using the GWAS cut-off of *P* < 1 × 10^−5^ vs. a GWAS cut-off of *P* < 1 × 10^−7^ indicates that using a more stringent GWAS threshold prevents the identification of any overlapping genes but has little impact on the number of identified GO terms (Fig. S3, Table S7, Supporting information).

## Discussion

It is widely accepted among gerontologists that common processes mechanistically underlie both aging and the pathogenesis of multiple age-related diseases and that targeting common factors in aging will have a significant benefit to human health (Berry & Cirulli, 2013; Torgovnick *et al*., 2013; Fontana *et al*., 2014; Sikora, 2014). A wealth of experimental data from lower organism studies supports this concept, and human progeroid syndromes indicate that disruption of key biological processes can result in the premature onset of multiple age-related pathologies (Ghosh & Zhou, 2014). There has, however, been little direct evidence that this is true in normal human aging and age-related disease, and the role of canonical aging pathways in human age-related pathologies has not been established.

Genomewide association studies, a common approach to identifying genetic loci of importance to human complex traits in large populations, have led to the discovery of key genes in a variety of individual age-related diseases such as Alzheimer’s disease (Kim *et al*., 2014), cardiovascular disease (Sayols-Baixeras *et al*., 2014), and a variety of age-associated cancers (Monteiro & Freedman, 2013; Barbieri & Tomlins, 2014; Veron *et al*., 2014), but GWAS meta-analyses have also yielded little in regards to identifying shared genes and pathways, identifying only APOE, 9P21, and the HLA loci (Jeck *et al*., 2012). The failure to identify canonical aging pathways by GWAS and GWAS meta-analyses has called into question whether these pathways are important to human longevity and health span. Determining whether age-related pathologies share common pathways is important given that current strategies aimed at developing interventions against age-related disease are based on the model that targeting underlying processes of aging can impact multiple diseases simultaneously.

Genomewide association studies approach has proven invaluable in identifying genes and regions of putative importance to human phenotypes and pathologies, but attempts to use GWAS to identify genetic variation that influences human longevity and health span have largely been unsuccessful (Newman *et al*., 2010) (Beekman *et al*., 2010) (Deelen *et al*., 2014). Among known genetic modifiers of human aging, only the APOE locus has been reproducibly associated with both lifespan and health span by GWAS (Deelen *et al*., 2011, 2014). The APOE locus is also strongly associated with neurological disease (particularly Alzheimer’s disease) (Naj *et al*., 2014; Wang *et al*., 2014), cardiac, metabolic, and vascular diseases (Maxwell *et al*., 2013; Hellwege *et al*., 2014), plasma C-reactive protein levels (Ellis *et al*., 2014; Schick *et al*., 2014), and, in a recent study, nonpathological cognitive aging (Davies *et al*., 2014). Thus, while GWAS have generally had difficulty in identifying genetic modulators of aging, apolipoprotein metabolism is an exception and appears to be a genuine GWAS detectable common pathway in human age-related disease. A more recent GWAS meta-analysis took a unique approach to studying aging by considering overall ‘wellness’, a surrogate for health span, based on the concept that successful aging would be related to broad resistance to disease (Jeck *et al*., 2012). These authors assessed bins of GWAS SNPs, by chromosome location, according to the number of unique diseases they are associated with in an attempt to identify genes common to many diseases and, by inference, general health span although, importantly, it should be noted that the analysis was inclusive of all disease-associated SNPs rather than considering only age-related diseases. This approach identified a set of regions associated with immune function, including the ubiquitous MHC locus, as well as the 9p21 senescence-associated locus, both also identified in our study (Fig.2), but failed to provide evidence supporting the role of conserved pathways such as IIS, mTOR, oxidative stress response, or genome maintenance.

Our gene-based findings suggest that while inflammation, immune regulation, and cholesterol metabolism are all broadly important in human aging, cholesterol metabolism genes alone are strikingly enriched among multiple age-related diseases. Multiple apolipoproteins have been associated with disease, and APOE is a particularly notable genetic loci in human health, as discussed. Consistent with these prior findings, our data suggest that apolipoprotein metabolism is a key underlying pathway in multiple human age-related diseases. Apolipoprotein genes have been associated with GWAS significance to a remarkable array of age-related pathologies including chronic renal disease, cardiovascular disease, inflammation, metabolic disease, hepatic dysfunction, alzheimer’s, dementia, and cognitive decline (Melegh *et al*., 2012; Schmidt *et al*., 2012; Wasser *et al*., 2012; Imes & Austin, 2013; Tosto & Reitz, 2013). Our findings suggest that apolipoprotein metabolism may represent a mammalian-specific underlying pathway in aging and age-related disease, supporting the notion that interventions in lipoprotein metabolism will provide significant benefits to human health. Epidemiological studies already support the adoption of earlier and more widespread statin use, and least one study has suggested that statins broadly affect the aging process (Boccardi *et al*., 2013; Robinson, 2014). Clearly, apolipoprotein metabolism warrants continued attention as a safe and efficacious clinical target in aging.

In addition to providing further evidence supporting the critical importance of apolipoprotein metabolism in human age-related disease, here, we provide evidence supporting for the model that common, evolutionarily conserved pathways influence many age-related diseases. The data presented here provide new evidence supporting the continued pursuit of interventions designed to combat age-related disease based on genetic pathways of aging discovered in lower organisms. While many of these pathways, such as genome maintenance and IIS/mTOR signaling, have already been implicated in human health, our study provides the first evidence that GWAS of age-related diseases show a signature of conserved pathways of aging. Finally, while our study focused on age-related disease, our novel pathway-based approach using gene ontology terms for comparison provides a new method for identifying shared pathways of disease. We anticipate that this approach can be applied to traits that are mechanistically poorly defined to provide novel insight into the pathogenesis of human diseases.

While our findings are supported by the previously published literature described our approach is not without limitations. The primary limitation of this study is the GWAS catalog itself—only SNPs identified in published GWAS at the significance level cut-off of *P* < 10^−5^ are included in this analysis. Therefore, our results only represent the portion of all genes important for age-related diseases that have been successfully identified by GWAS. An additional caveat of our approach is that the initial step of assigning GWAS traits to disease and age-related vs. non-age-related categories introduces some interpretation in the analysis. While each subsequent step is completely unbiased differences in trait assignment may slightly affect the result. Fortunately, given the scarcity of overlapping genes and the weak impact that decreasing the *P*-value cut-off to *P* < 10^−7^ has on the identified overlapping GO terms, it appears that minor alterations to trait assignment will have no major impact on the outcome using this approach. Finally, while our approach involved a GWAS *P*-value cut-off in the initial stage of analysis, it may also be possible to examine all GWAS SNPs for association with multiple age-related disease categories and rank them by Fisher’s combined probability test *P*-value. This approach would likely identify additional associations.

## Methods

### GWAS data and associated genes

The GWAS catalog was downloaded from the National Human Genome Research Institute (http://www.genome.gov/26525384) (Welter *et al*., 2014). At the time of download on November 9, 2013, the catalog contained 1738 GWAS, reporting 11 533 SNPs with assigned ‘rs’ numbers associated with 816 diseases/traits. To prevent bias due to limited numbers of studies for some diseases/traits, we limited our analyses to those with at least 5 independent GWAS reports resulting in 39 age-related diseases. This dataset includes a total of 410 independent GWAS (Table1). SNPs were assigned to genes based if they are located on a gene or they are in high linkage disequilibrium (LD) with SNPs on a gene. The LD information is calculated using SCAN database (http://www.scandb.org/newinterface/about.html) (Gamazon *et al*., 2010). These assignments produced 1975 protein-coding genes in high or complete LD or overlap with the diseases/trait-associated SNPs. Individual traits were assigned to one of five age-related disease categories, or determined to be non-age-associated, based on established criteria (see Results and (Perez-Lopez *et al*., 2009; Martin, 2012; Johnson *et al*., 2013; Brunet & Berger, 2014)).

### GO enrichment analysis and visualization

Gene ontology (GO) analysis was performed using the Gene Ontology enRIchment anaLysis and visuaLizAtion tool (GORILLA) (http://cbl-gorilla.cs.technion.ac.il/) (Eden *et al*., 2009), with graphical representations of gene ontology trees and enrichment *P*-values produced using the linked REViGO tool (http://revigo.irb.hr/) (Supek *et al*., 2011). Genes appearing 3 or more categories were analyzed against the background set of the entire gene list using the two unranked lists of genes analysis mode. Visualizations of GO terms found in the overlap between all age-related disease groups (Figs4 and S2) were produced using REViGO.

### GO term similarity measurement

Given that standard gene ontology enrichment analyses cannot be performed on this data, as there is no standardized approach to determining pathway enrichment from a GO term set (GO term enrichment is typically measured against a gene set), we considered the statistical value of this result using an *ad hoc* assessment. If the GO terms appearing in the overlapping term set represents an enrichment of specific processes, then we would expect the GO terms to be closely related as defined by their proximity in an ontology tree (Wang *et al*., 2007). Briefly, we (i) aggregated the contributions of each GO term based on ancestor terms in the gene ontology tree to give a semantic value for the term; and (ii) we calculated the semantic similarity of each pair of GO terms based on these values (detailed method in (Wang *et al*., 2007)). Adopting this method, pairwise similarities of GO terms in the overlap between the five disease groups, and in the background set, were estimated separately using an R package ‘GOSemSim’ based on GO biological process tree (Yu *et al*., 2010). The significance of the mean value difference between the GO terms in the disease group overlap compared to the background set was determined using one-sided Wilcoxon rank-sum test.

### *P*-value calculation in pathway-based approach

We calculated the pairwise similarity of this GO term group and compared it to the baseline set. The calculated similarity for the overlapping set was determined to be 0.15 ± 0.13, with the background set similarity calculated as 0.13 ± 0.1 (*P*-value is < 1E-15, one-sided Wilcoxon rank-sum test), indicating that the relative similarity of GO terms in the overlapping set could not be achieved by random selection.

## Supporting Information

**Fig. S1** Enriched Biological Process GO terms in genes associated with multiple age-related diseases. Gene ontology analysis revealed cholesterol metabolism ontology terms as highly and specifically enriched among genes associated with multiple age-related disease categories by GWAS (cellular component terms in Fig. ).

**Fig. S2** Principal component analysis of gene ontology terms appearing in all 5 age-related disease categories. A principal component analysis plotted using REViGO. Color indicates term uniqueness while size represents the log size of the term group in the gene ontology tree. Key parent terms are indicated.

**Fig. S3** Venn Diagrams of Genes and SNPs in Age-Related Disease Categories using an Initial GWAS Cut-off of *P* < 10^−7^.

**Table S1** GWAS traits not meeting our criteria for study number and statistical strength but not included in any of the 5 age-related disease categories.

**Table S2** Enriched Gene Ontology Terms In Genes Shared by 3 or More Age-related Disease Groups. False discovery *P*-value is calculated using the Benjamani–Hochberg method.

**Table S3** Select GO terms appearing in all 5 age-related disease categories. A full list of the 209 shared terms is provided in Table S4.

**Table S4** A full list of enriched GO terms found in the overlapping term set.

**Table S5** Genes Associated with the GWAS Trait Longevity at *P* < 1 × 10^−5^. See Table2 for comparison with genes associated with multiple age-related diseases. Only APOE and APOCI are common between these two gene sets.

**Table S6** Biological Process GO Terms Associated with Longevity GWAS Trait. Only GO terms with nominal *P*-value < 0.05 listed here. No GO terms remain significant following Benjamini multiple testing correction. GO terms associated with apolipoprotein metabolism indicated in bold. See Table S2 and S3 for comparison with GO terms found in all 5 age-related disease groups.

**Table S7** Comparison of SNP, Gene, and GO Term Numbers Identified by GWAS Cut-off
