# Supplementary figures and images for: Genetic evidence for common pathways in human age-related diseases
Source: Aging Cell. 2015 Jun 15;14(5):809–17. doi: 10.1111/acel.12362 (PMC4568968; doi:10.1111/acel.12362)

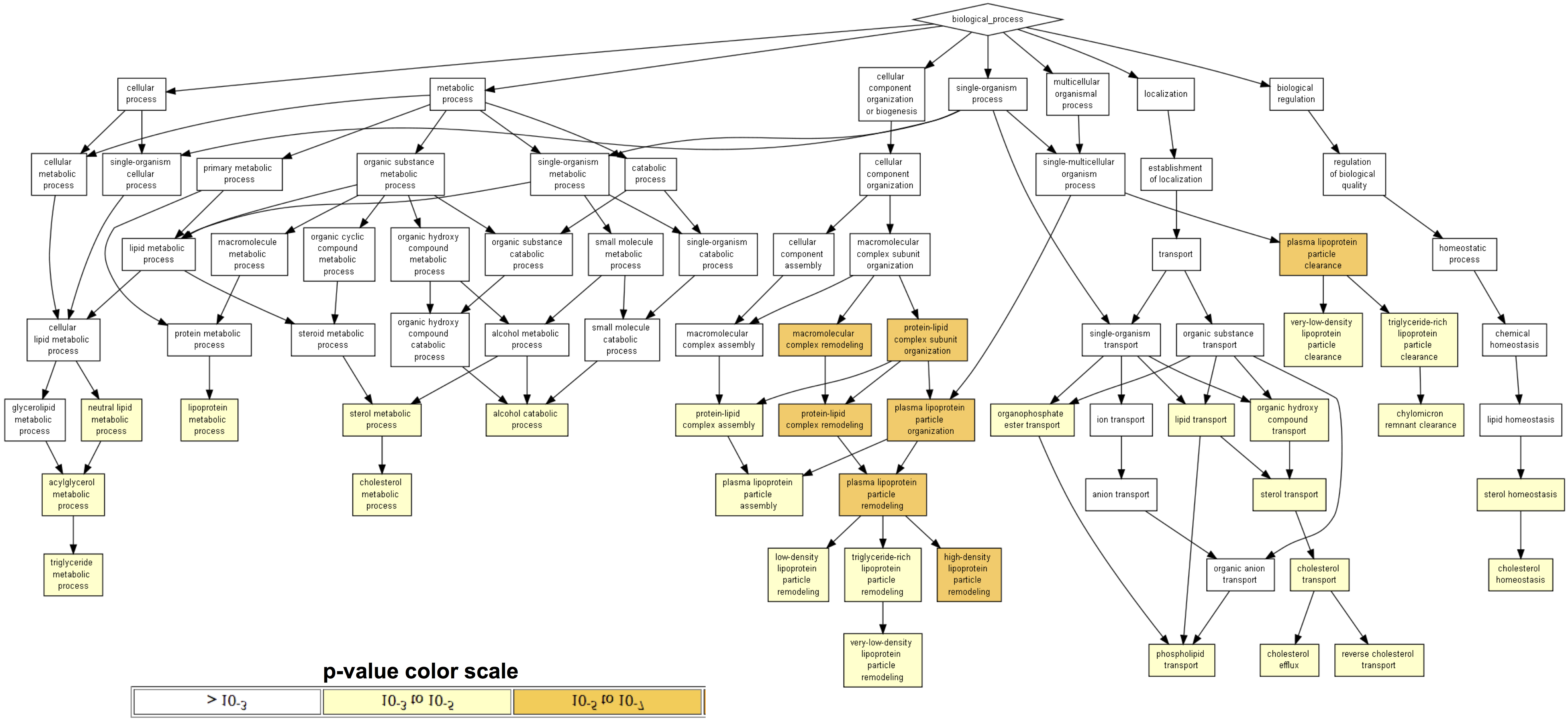

Supplement: Supplementary file 1 [file acel0014-0809-sd1.tiff]

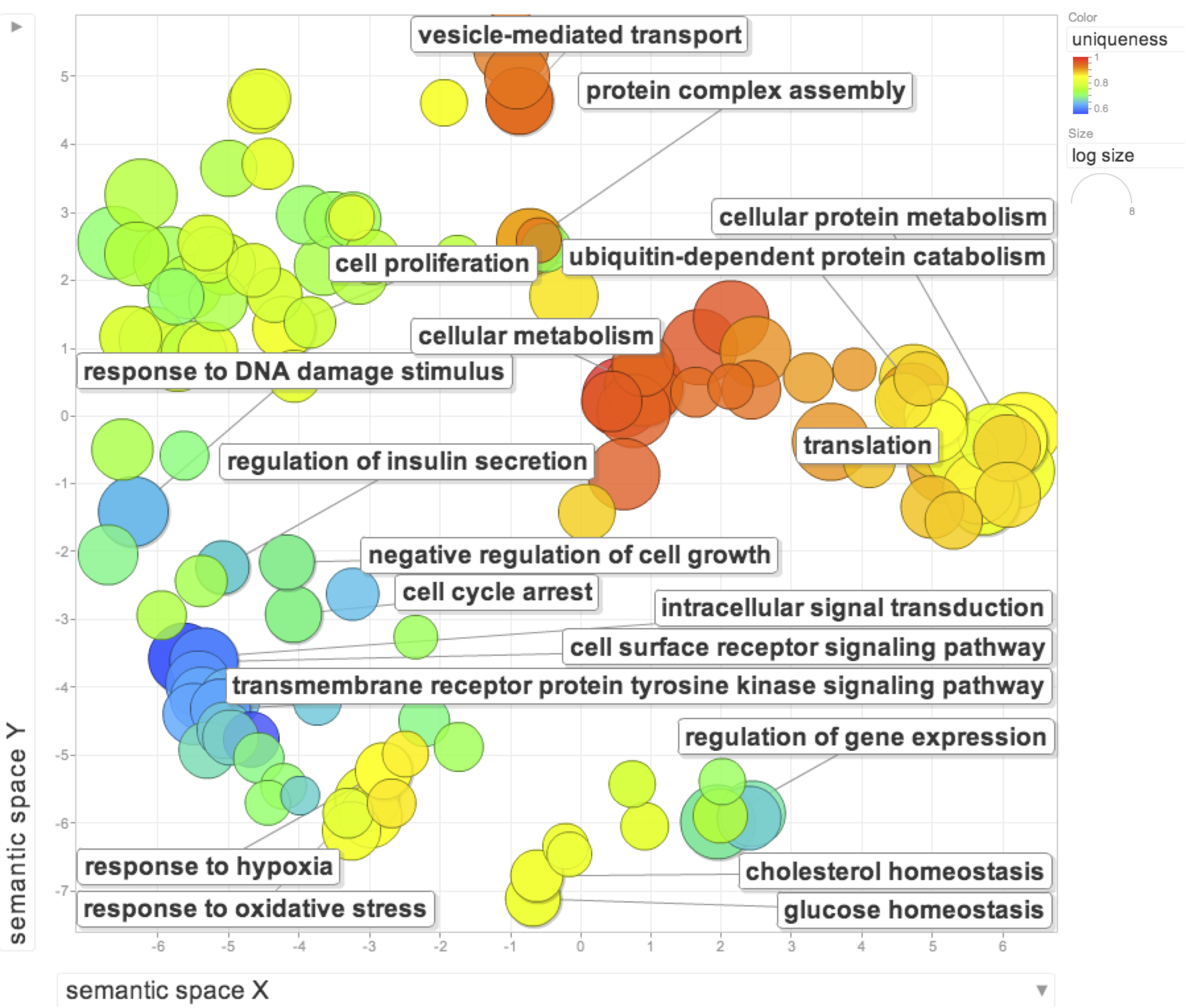

Supplement: Supplementary file 2 [file acel0014-0809-sd2.tiff]

A

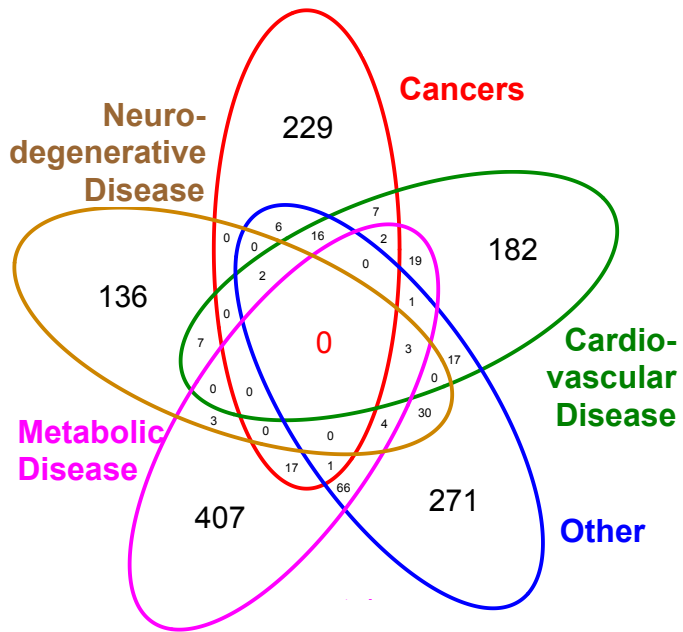

Unique objects: All = 1426; S1 = 0; S2 = 0; S3 = 0; S4 = 0; S5 = 0

B

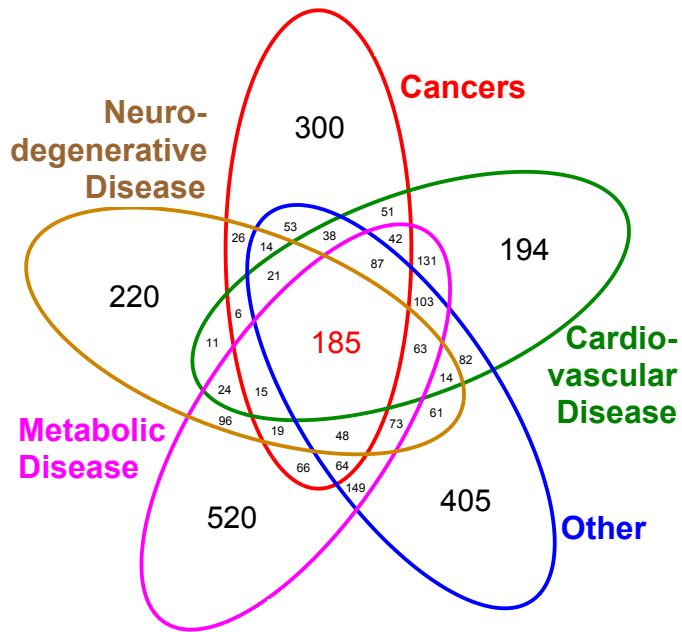

Unique objects: All = 3181; S1 = 0; S2 = 0; S3 = 0; S4 = 0; S5 = 0

Supplement: Supplementary file 3 [file acel0014-0809-sd3.pdf]
