# Supplementary material for: Genetic evidence for common pathways in human age-related diseases
Source: Aging Cell. 2015 Jun 15;14(5):809–17. doi: 10.1111/acel.12362 (PMC4568968; doi:10.1111/acel.12362)
Supplement: Supplementary file 4 [file acel0014-0809-sd4.docx]

| **Table S1 – GWAS traits not meeting our criteria for study number and statistical strength but not included in any of the 5 age-related disease categories.** | | |
| --- | --- | --- |
| **Disease** | **Number of GWAS** | **Significant SNPs** |
| Acute lymphoblastic leukemia (childhood) | 5 | 37 |
| Alcohol dependence | 8 | 23 |
| Asthma | 14 | 53 |
| Attention deficit hyperactivity disorder | 6 | 66 |
| Autism | 5 | 9 |
| Bilirubin levels | 6 | 27 |
| Bipolar disorder | 15 | 119 |
| Crohn's disease | 14 | 173 |
| Height | 21 | 461 |
| Kawasaki disease | 6 | 19 |
| Major depressive disorder | 9 | 75 |
| Menarche (age at onset) | 6 | 65 |
| Orofacial clefts | 5 | 45 |
| Psoriasis | 7 | 38 |
| Response to antipsychotic treatment | 5 | 57 |
| Response to hepatitis C treatment | 6 | 15 |
| Schizophrenia | 21 | 111 |
| Smoking behavior | 7 | 41 |
| Systemic lupus erythematosus | 11 | 90 |
| Type 1 diabetes | 10 | 78 |
| Vitiligo | 6 | 37 |
| Ulcerative colitis | 10 | 115 |
|  |  |  |
