# Supplementary material for: Genetic evidence for common pathways in human age-related diseases
Source: Aging Cell. 2015 Jun 15;14(5):809–17. doi: 10.1111/acel.12362 (PMC4568968; doi:10.1111/acel.12362)
Supplement: Supplementary file 5 [file acel0014-0809-sd5.docx]

| **Table S2: Enriched Gene Ontology Terms In Genes Shared by 3 or More Age-related Disease Groups.** | | | | | | | | | |
| --- | --- | --- | --- | --- | --- | --- | --- | --- | --- |
| **GO Term** | **Description** | **p-value** | **FDR p-value** | **Enrichment** | **N** | **B** | **n** | **b** | **Genes** |
| **Cellular Component** | |  |  |  |  |  |  |  |  |
| GO:0034364 | high-density lipoprotein particle | 2.24E-06 | 1.83E-03 | 20.25 | 1620 | 16 | 25 | 5 | LIPC, APOM, APOE, CETP, APOC1 |
| GO:0034358 | plasma lipoprotein particle | 7.63E-06 | 3.11E-03 | 16.2 | 1620 | 20 | 25 | 5 | LIPC, APOM, APOE, CETP, APOC1 |
| GO:0032994 | protein-lipid complex | 9.91E-06 | 2.69E-03 | 15.43 | 1620 | 21 | 25 | 5 | LIPC, APOM, APOE, CETP, APOC1 |
| GO:0034361 | very-low-density lipoprotein particle | 2.57E-04 | 5.24E-02 | 21.6 | 1620 | 9 | 25 | 3 | APOM, APOE, APOC1 |
| GO:0034385 | triglyceride-rich lipoprotein particle | 2.57E-04 | 4.19E-02 | 21.6 | 1620 | 9 | 25 | 3 | APOM, APOE, APOC1 |
| **Biological Process** | |  |  |  |  |  |  |  |  |
| GO:0034375 | high-density lipoprotein particle remodeling | 2.50E-07 | 1.80E-03 | 29.45 | 1620 | 11 | 25 | 5 | LIPC, APOM, APOE, CETP, APOC1 |
| GO:0034367 | macromolecular complex remodeling | 1.05E-06 | 3.78E-03 | 23.14 | 1620 | 14 | 25 | 5 | LIPC, APOM, APOE, CETP, APOC1 |
| GO:0034368 | protein-lipid complex remodeling | 1.05E-06 | 2.52E-03 | 23.14 | 1620 | 14 | 25 | 5 | LIPC, APOM, APOE, CETP, APOC1 |
| GO:0034369 | plasma lipoprotein particle remodeling | 1.05E-06 | 1.89E-03 | 23.14 | 1620 | 14 | 25 | 5 | LIPC, APOM, APOE, CETP, APOC1 |
| GO:0071827 | plasma lipoprotein particle organization | 2.24E-06 | 3.23E-03 | 20.25 | 1620 | 16 | 25 | 5 | LIPC, APOM, APOE, CETP, APOC1 |
| GO:0071825 | protein-lipid complex subunit organization | 3.14E-06 | 3.77E-03 | 19.06 | 1620 | 17 | 25 | 5 | LIPC, APOM, APOE, CETP, APOC1 |
| GO:0034381 | plasma lipoprotein particle clearance | 8.73E-06 | 8.98E-03 | 25.92 | 1620 | 10 | 25 | 4 | LIPC, APOM, APOE, CETP, APOC1 |
| GO:0030301 | cholesterol transport | 1.27E-05 | 1.14E-02 | 14.73 | 1620 | 22 | 25 | 5 | LIPC, APOM, APOE, CETP, APOC1 |
| GO:0015918 | sterol transport | 1.27E-05 | 1.02E-02 | 14.73 | 1620 | 22 | 25 | 5 | LIPC, APOM, APOE, CETP, APOC1 |
| GO:0071830 | triglyceride-rich lipoprotein particle clearance | 1.29E-05 | 9.27E-03 | 48.6 | 1620 | 4 | 25 | 3 | LIPC, APOE, APOC1 |
| GO:0034374 | low-density lipoprotein particle remodeling | 1.29E-05 | 8.43E-03 | 48.6 | 1620 | 4 | 25 | 3 | LIPC, APOE, APOC1 |
| GO:0034382 | chylomicron remnant clearance | 1.29E-05 | 7.73E-03 | 48.6 | 1620 | 4 | 25 | 3 | LIPC, APOE, APOC1 |
| GO:0043691 | reverse cholesterol transport | 2.01E-05 | 1.12E-02 | 21.6 | 1620 | 12 | 25 | 4 | LIPC, APOE, CETP |
| GO:0006641 | triglyceride metabolic process | 3.99E-05 | 2.05E-02 | 18.51 | 1620 | 14 | 25 | 4 | LIPC, APOE, CETP, APOC1 |
| GO:0015850 | organic hydroxy compound transport | 5.32E-05 | 2.55E-02 | 11.17 | 1620 | 29 | 25 | 5 | LIPC, APOE, CETP, APOC1 |
| GO:0034372 | very-low-density lipoprotein particle remodeling | 6.31E-05 | 2.84E-02 | 32.4 | 1620 | 6 | 25 | 3 | LIPC, APOE, CETP |
| GO:0042157 | lipoprotein metabolic process | 7.10E-05 | 3.01E-02 | 16.2 | 1620 | 16 | 25 | 4 | APOM, APOE, CETP, APOC1 |
| GO:0006639 | acylglycerol metabolic process | 9.19E-05 | 3.68E-02 | 15.25 | 1620 | 17 | 25 | 4 | LIPC, APOE, CETP, APOC1 |
| GO:0034370 | triglyceride-rich lipoprotein particle remodeling | 1.09E-04 | 4.14E-02 | 27.77 | 1620 | 7 | 25 | 3 | LIPC, APOE, CETP |
| GO:0006638 | neutral lipid metabolic process | 1.17E-04 | 4.21E-02 | 14.4 | 1620 | 18 | 25 | 4 | LIPC, APOE, CETP, APOC1 |
| GO:0034447 | very-low-density lipoprotein particle clearance | 2.29E-04 | 7.85E-02 | 64.8 | 1620 | 2 | 25 | 2 | APOE, APOC1 |
| GO:0006869 | lipid transport | 2.32E-04 | 7.61E-02 | 8.31 | 1620 | 39 | 25 | 5 | LIPC, APOM, APOE, APOC1, CETP |
| GO:0034377 | plasma lipoprotein particle assembly | 2.57E-04 | 8.04E-02 | 21.6 | 1620 | 9 | 25 | 3 | APOM, APOE, APOC1 |
| GO:0065005 | protein-lipid complex assembly | 3.63E-04 | 1.09E-01 | 19.44 | 1620 | 10 | 25 | 3 | APOM, APOE, APOC1 |
| GO:0055092 | sterol homeostasis | 3.81E-04 | 1.10E-01 | 10.8 | 1620 | 24 | 25 | 4 | LIPC, APOM, APOE, CETP |
| GO:0042632 | cholesterol homeostasis | 3.81E-04 | 1.06E-01 | 10.8 | 1620 | 24 | 25 | 4 | LIPC, APOM, APOE, CETP |
| GO:0008203 | cholesterol metabolic process | 5.26E-04 | 1.40E-01 | 9.97 | 1620 | 26 | 25 | 4 | LIPC, APOM, APOE, CETP |
| GO:0015914 | phospholipid transport | 6.52E-04 | 1.68E-01 | 16.2 | 1620 | 12 | 25 | 3 | APOE, APOC1, CETP |
| GO:0046164 | alcohol catabolic process | 6.52E-04 | 1.62E-01 | 16.2 | 1620 | 12 | 25 | 3 | LIPC, APOE, ALDH2 |
| GO:0016125 | sterol metabolic process | 8.09E-04 | 1.94E-01 | 8.94 | 1620 | 29 | 25 | 4 | LIPC, APOC1, CETP |
| GO:0015748 | organophosphate ester transport | 8.39E-04 | 1.95E-01 | 14.95 | 1620 | 13 | 25 | 3 | APOE, APOC1, CETP |
| GO:0033344 | cholesterol efflux | 8.39E-04 | 1.89E-01 | 14.95 | 1620 | 13 | 25 | 3 | APOM, APOE, APOC1 |
| False discovery p-value is calculated using the Benjamani- Hochberg method. | | | | | |  |  |  |  |
