# Supplementary material for: Genetic evidence for common pathways in human age-related diseases
Source: Aging Cell. 2015 Jun 15;14(5):809–17. doi: 10.1111/acel.12362 (PMC4568968; doi:10.1111/acel.12362)
Supplement: Supplementary file 6 [file acel0014-0809-sd6.docx]

| **Table S3 – Select GO terms appearing in all 5 age-related disease categories.** | | | |
| --- | --- | --- | --- |
|  | **GO Term ID** | **Description** | |
| **Signaling/Signal Transduction** | GO:0007169 | Transmembrane receptor protein tyrosine kinase signaling pathway |  |
|  | GO:0007166 | Cell surface receptor signaling pathway |  |
|  | GO:0048015 | Phosphatidylinositol-mediated signaling |  |
|  | GO:0035556 | Intracellular signal transduction |  |
|  | GO:0007165 | Signal transduction |  |
|  | GO:0000165 | MAPK cascade |  |
|  | GO:0043123 | Positive regulation of I-kappaB kinase/NF-kappaB cascade |  |
|  | GO:0051897 | Positive regulation of protein kinase B signaling cascade |  |
| **Translation** |  |  |  |
|  | GO:0006412 | Translation |  |
|  | GO:0016071 | mRNA metabolic process |  |
|  |  |  |  |
| **Stress Responses (DNA damage, oxidative stress, etc)** | GO:0006979 | Response to oxidative stress |  |
|  | GO:0006974 | Response to DNA damage stimulus |  |
|  | GO:0006281 | DNA repair |  |
|  | GO:0001666 | Response to hypoxia |  |
|  | GO:0006464 | Cellular protein modification process |  |
|  | GO:0006468 | Protein phosphorylation |  |
|  | GO:0031648 | Protein destabilization |  |
|  | GO:0050821 | Protein stabilization |  |
|  | GO:0043687 | Post-translational protein modification |  |
|  | GO:0006626 | Protein targeting to mitochondrion |  |
|  | GO:0044267 | Cellular protein metabolic process |  |
|  | GO:0006511 | Ubiquitin-dependent protein catabolic process |  |
|  | GO:0016567 | Protein ubiquitination |  |
|  |  |  |  |
| **Glucose/Cholesterol Homeostasis** | GO:0042593 | Glucose homeostasis |  |
|  | GO:0044255 | Cellular lipid metabolic process |  |
|  | GO:0042632 | Cholesterol homeostasis |  |
|  | GO:0043691 | Reverse cholesterol transport |  |
|  | GO:0033344 | Cholesterol efflux |  |
|  | GO:0032374 | Regulation of cholesterol transport |  |
|  | GO:0034384 | High-density lipoprotein particle clearance |  |
|  | GO:0034375 | High-density lipoprotein particle remodeling |  |
|  | GO:0034380 | High-density lipoprotein particle assembly |  |
|  |  |  |  |
| **Inflammation** | GO:0043433 | Negative regulation of sequence-specific DNA binding transcription factor activity |  |
|  | GO:0051092 | Positive regulation of NF-kappaB transcription factor activity |  |
|  | GO:0002455 | Humoral immune response mediated by circulating immunoglobulin |  |
|  | GO:0045954 | Positive regulation of natural killer cell mediated cytotoxicity |  |
|  | GO:0002381 | Immunoglobulin production involved in immunoglobulin mediated immune response |  |
|  | GO:0006968 | Cellular defense response |  |
|  | GO:0006954 | Inflammatory response |  |
|  |  |  |  |
| **Cell Fate (Cell cycle arrest, apoptosis, etc)** | GO:0045165 | Cell fate commitment |  |
|  | GO:0006338 | Chromatin remodeling |  |
|  | GO:0000086 | G2/M transition of mitotic cell cycle |  |
|  | GO:0000082 | G1/S transition of mitotic cell cycle |  |
|  | GO:0010468 | Regulation of gene expression |  |
|  | GO:0007050 | Cell cycle arrest |  |
|  | GO:0050796 | Regulation of insulin secretion |  |
|  | GO:0008283 | Cell proliferation |  |
|  | GO:0006915 | Apoptotic process |  |
|  | GO:0030308 | Negative regulation of cell growth |  |
| A full list of the 209 shared terms is provided in Table S3. | | |  |
