# Supplementary material for: Genetic evidence for common pathways in human age-related diseases
Source: Aging Cell. 2015 Jun 15;14(5):809–17. doi: 10.1111/acel.12362 (PMC4568968; doi:10.1111/acel.12362)
Supplement: Supplementary file 7 [file acel0014-0809-sd7.pdf]

GO ID	GO Term Name
GO:0008150	biological_process
GO:0044237	cellular metabolic process
GO:0034641	cellular nitrogen compound metabolic process
GO:0044281	small molecule metabolic process
GO:0010467	gene expression
GO:0006810	transport
GO:0055114	oxidation-reduction process
GO:0016070	RNA metabolic process
GO:0005975	carbohydrate metabolic process
GO:0006811	ion transport
GO:0006508	proteolysis
GO:0016032	viral reproduction
GO:0006396	RNA processing
GO:0002474	antigen processing and presentation of peptide antigen via MHC class I
GO:0002479	antigen processing and presentation of exogenous peptide antigen via MHC class I, TAP-dependent
GO:0042590	antigen processing and presentation of exogenous peptide antigen via MHC class I
GO:0050776	regulation of immune response
GO:0019886	antigen processing and presentation of exogenous peptide antigen via MHC class II
GO:0045087	innate immune response
GO:0006955	immune response
GO:0007169	transmembrane receptor protein tyrosine kinase signaling pathway
GO:0008543	fibroblast growth factor receptor signaling pathway
GO:0048011	neurotrophin TRK receptor signaling pathway
GO:0007173	epidermal growth factor receptor signaling pathway
GO:0008286	insulin receptor signaling pathway
GO:0030511	positive regulation of transforming growth factor beta receptor signaling pathway
GO:0031648	protein destabilization
GO:0050821	protein stabilization
GO:0006412	translation
GO:0006979	response to oxidative stress
GO:0042593	glucose homeostasis
GO:0006879	cellular iron ion homeostasis
GO:0006974	response to DNA damage stimulus
GO:0006281	DNA repair
GO:0035556	intracellular signal transduction
GO:0007165	signal transduction
GO:0016567	protein ubiquitination
GO:0001934	positive regulation of protein phosphorylation
GO:0050731	positive regulation of peptidyl-tyrosine phosphorylation
GO:0051436	negative regulation of ubiquitin-protein ligase activity involved in mitotic cell cycle
GO:0051437	positive regulation of ubiquitin-protein ligase activity involved in mitotic cell cycle
GO:0031398	positive regulation of protein ubiquitination
GO:0051439	regulation of ubiquitin-protein ligase activity involved in mitotic cell cycle
GO:0000079	regulation of cyclin-dependent protein serine/threonine kinase activity
GO:0070936	protein K48-linked ubiquitination
GO:0000187	activation of MAPK activity
GO:0001666	response to hypoxia
GO:0045165	cell fate commitment
GO:0044255	cellular lipid metabolic process
GO:0008283	cell proliferation
GO:0006968	cellular defense response
GO:0042060	wound healing
GO:0050728	negative regulation of inflammatory response
GO:0007596	blood coagulation
GO:0030168	platelet activation
GO:0006954	inflammatory response
GO:0017144	drug metabolic process
GO:0034063	stress granule assembly
GO:0042632	cholesterol homeostasis
GO:0007220	Notch receptor processing
GO:0006112	energy reserve metabolic process
GO:0001523	retinoid metabolic process
GO:0030203	glycosaminoglycan metabolic process
GO:0006027	glycosaminoglycan catabolic process
GO:0008380	RNA splicing
GO:0007605	sensory perception of sound
GO:0007202	activation of phospholipase C activity
GO:0000278	mitotic cell cycle
GO:0007267	cell-cell signaling
GO:0071277	cellular response to calcium ion
GO:0043691	reverse cholesterol transport
GO:0033344	cholesterol efflux
GO:0032374	regulation of cholesterol transport
GO:0006928	cellular component movement
GO:0006367	transcription initiation from RNA polymerase II promoter
GO:0006357	regulation of transcription from RNA polymerase II promoter
GO:0000122	negative regulation of transcription from RNA polymerase II promoter
GO:0006915	apoptotic process
GO:0043065	positive regulation of apoptotic process
GO:0043066	negative regulation of apoptotic process
GO:0042981	regulation of apoptotic process
GO:0006919	activation of cysteine-type endopeptidase activity involved in apoptotic process
GO:0016477	cell migration
GO:0007155	cell adhesion
GO:0006898	receptor-mediated endocytosis
GO:0006198	cAMP catabolic process
GO:0030819	positive regulation of cAMP biosynthetic process
GO:0000184	nuclear-transcribed mRNA catabolic process, nonsense-mediated decay
GO:0016071	mRNA metabolic process
GO:0031532	actin cytoskeleton reorganization
GO:0007263	nitric oxide mediated signal transduction
GO:0019933	cAMP-mediated signaling
GO:0006366	transcription from RNA polymerase II promoter
GO:0050995	negative regulation of lipid catabolic process
GO:0019369	arachidonic acid metabolic process
GO:0016601	Rac protein signal transduction
GO:0030198	extracellular matrix organization
GO:0022617	extracellular matrix disassembly
GO:0045216	cell-cell junction organization
GO:0034329	cell junction assembly
GO:0034332	adherens junction organization
GO:0045893	positive regulation of transcription, DNA-dependent
GO:0045944	positive regulation of transcription from RNA polymerase II promoter
GO:0001819	positive regulation of cytokine production
GO:0007219	Notch signaling pathway
GO:0008202	steroid metabolic process
GO:0006816	calcium ion transport
GO:0048015	phosphatidylinositol-mediated signaling
GO:0006821	chloride transport
GO:0034384	high-density lipoprotein particle clearance
GO:0034375	high-density lipoprotein particle remodeling
GO:0034380	high-density lipoprotein particle assembly
GO:0048662	negative regulation of smooth muscle cell proliferation
GO:0016044	cellular membrane organization
GO:0016045	detection of bacterium
GO:0030574	collagen catabolic process
GO:0033137	negative regulation of peptidyl-serine phosphorylation
GO:0038095	Fc-epsilon receptor signaling pathway
GO:0050852	T cell receptor signaling pathway
GO:0038096	Fc-gamma receptor signaling pathway involved in phagocytosis
GO:0043433	negative regulation of sequence-specific DNA binding transcription factor activity
GO:0051092	positive regulation of NF-kappaB transcription factor activity
GO:0006184	GTP catabolic process
GO:0000165	MAPK cascade
GO:0043123	positive regulation of I-kappaB kinase/NF-kappaB cascade
GO:0051897	positive regulation of protein kinase B signaling cascade
GO:0030308	negative regulation of cell growth
GO:0007166	cell surface receptor signaling pathway
GO:0002455	humoral immune response mediated by circulating immunoglobulin
GO:0045954	positive regulation of natural killer cell mediated cytotoxicity
GO:0002381	immunoglobulin production involved in immunoglobulin mediated immune response
GO:0006338	chromatin remodeling
GO:0006464	cellular protein modification process
GO:0006468	protein phosphorylation
GO:0000086	G2/M transition of mitotic cell cycle
GO:0000082	G1/S transition of mitotic cell cycle
GO:0060070	canonical Wnt receptor signaling pathway
GO:0090263	positive regulation of canonical Wnt receptor signaling pathway
GO:0006418	tRNA aminoacylation for protein translation
GO:0051000	positive regulation of nitric-oxide synthase activity
GO:0035690	cellular response to drug
GO:0043687	post-translational protein modification
GO:0006626	protein targeting to mitochondrion
GO:0034220	ion transmembrane transport
GO:0001709	cell fate determination
GO:0007271	synaptic transmission, cholinergic
GO:0006461	protein complex assembly
GO:0051262	protein tetramerization
GO:0007264	small GTPase mediated signal transduction
GO:0006351	transcription, DNA-dependent
GO:0006355	regulation of transcription, DNA-dependent
GO:0046777	protein autophosphorylation
GO:0002576	platelet degranulation
GO:0009615	response to virus
GO:0016525	negative regulation of angiogenesis
GO:0007156	homophilic cell adhesion
GO:0010468	regulation of gene expression
GO:0031295	T cell costimulation
GO:0042130	negative regulation of T cell proliferation
GO:0042102	positive regulation of T cell proliferation
GO:0006936	muscle contraction
GO:0044267	cellular protein metabolic process
GO:0006511	ubiquitin-dependent protein catabolic process
GO:0031145	anaphase-promoting complex-dependent proteasomal ubiquitin-dependent protein catabolic process
GO:0006805	xenobiotic metabolic process
GO:0019221	cytokine-mediated signaling pathway
GO:0060337	type I interferon-mediated signaling pathway
GO:0060333	interferon-gamma-mediated signaling pathway
GO:0030879	mammary gland development
GO:0016055	Wnt receptor signaling pathway
GO:0007186	G-protein coupled receptor signaling pathway
GO:0007010	cytoskeleton organization
GO:0042147	retrograde transport, endosome to Golgi
GO:0007603	phototransduction, visible light
GO:0002224	toll-like receptor signaling pathway
GO:0048146	positive regulation of fibroblast proliferation
GO:0050679	positive regulation of epithelial cell proliferation
GO:0008284	positive regulation of cell proliferation
GO:0008285	negative regulation of cell proliferation
GO:0009653	anatomical structure morphogenesis
GO:0007399	nervous system development
GO:0007275	multicellular organismal development
GO:0030154	cell differentiation
GO:0009790	embryo development
GO:0050796	regulation of insulin secretion
GO:0007268	synaptic transmission
GO:0016192	vesicle-mediated transport
GO:0045892	negative regulation of transcription, DNA-dependent
GO:0007265	Ras protein signal transduction
GO:0051056	regulation of small GTPase mediated signal transduction
GO:0001822	kidney development
GO:0007409	axonogenesis
GO:0007411	axon guidance
GO:0030097	hemopoiesis
GO:0055010	ventricular cardiac muscle tissue morphogenesis
GO:0031175	neuron projection development
GO:0007517	muscle organ development
GO:0048699	generation of neurons
GO:0007417	central nervous system development
GO:0007420	brain development
GO:0055085	transmembrane transport
GO:0050900	leukocyte migration
GO:0030335	positive regulation of cell migration
GO:0030336	negative regulation of cell migration
GO:0043537	negative regulation of blood vessel endothelial cell migration
GO:0006309	apoptotic DNA fragmentation
GO:0007050	cell cycle arrest
GO:0001501	skeletal system development
GO:0007601	visual perception

Table S3: A full list of enriched GO terms found in the overlapping term set.
