# Supplementary material for: Genetic evidence for common pathways in human age-related diseases
Source: Aging Cell. 2015 Jun 15;14(5):809–17. doi: 10.1111/acel.12362 (PMC4568968; doi:10.1111/acel.12362)
Supplement: Supplementary file 8 [file acel0014-0809-sd8.pdf]

Table S5 – Genes Associated with the GWAS Trait Longevity at p<1x10-5

Gene	Encoded Protein
ABCC4	ATP-binding cassette, sub-family C (CFTR/MRP), member 4
ADGRD1	G protein-coupled receptor 133
AKR1B10	Aldo-keto reductase family 1, member B10 (aldose reductase)
APOC1	Apolipoprotein C-I
APOE	Apolipoprotein E
ARSA	Arylsulfatase A
ATRIP	ATR interacting protein
C1QTNF6	C1q and tumor necrosis factor related protein 6
C7orf50	Chromosome 7 open reading frame 50
CAMK4	Calcium/calmodulin-dependent protein kinase IV
CARS	Cysteinyl-tRNA synthetase
CCDC51	Coiled-coil domain containing 51
CDH4	Cadherin 4, type 1, R-cadherin (retinal)
COL5A1	Collagen, type V, alpha 1
CXCL10	Chemokine (C-X-C motif) ligand 10
CXCL11	Chemokine (C-X-C motif) ligand 11
CXCL9	Chemokine (C-X-C motif) ligand 9
FBXW12	F-box and WD repeat domain containing 12
GABRB3	Gamma-aminobutyric acid (GABA) A receptor, beta 3
MAD1L1	MAD1 mitotic arrest deficient-like 1 (yeast)
MAEA	Macrophage erythroblast attacher
NAAA	N-acylethanolamine acid amidase
NCAM2	Neural cell adhesion molecule 2
NRXN1	Neurexin 1
NTHL1	Nth endonuclease III-like 1 (E. coli)
PFKFB4	Proline rich 18
PLXNB1	Plexin B1
PRR18	6-phosphofructo-2-kinase/fructose-2,6-biphosphatase 4
PSMC1	Proteasome (prosome, macropain) 26S subunit, ATPase, 1
PVRL2	Poliovirus receptor-related 2 (herpesvirus entry mediator B)
RAC2	Ras-related C3 botulinum toxin substrate 2
SDAD1	SDA1 domain containing 1
SFT2D1	SFT2 domain containing 1
SHISA5	Shisa homolog 5 (Xenopus laevis)
SLC38A10	Solute carrier family 38, member 10
SPINK8	Serine peptidase inhibitor, Kazal type 8 (putative)
SSTR3	Somatostatin receptor 3
STK24	Serine/threonine kinase 24 (STE20 homolog, yeast)
SVEP1	Sushi, von Willebrand factor type A, EGF and pentraxin domain containing 1
