# Supplementary material for: Genetic evidence for common pathways in human age-related diseases
Source: Aging Cell. 2015 Jun 15;14(5):809–17. doi: 10.1111/acel.12362 (PMC4568968; doi:10.1111/acel.12362)
Supplement: Supplementary file 9 [file acel0014-0809-sd9.pdf]

Table S6 – Biological Process GO Terms Associated with Longevity GWAS Trait

GO Term	Gene Count	Enrichment P-Value	Benjamini Corrected P-value
Taxis	5	0.0011	0.55
Chemotaxis	5	0.0011	0.55
Cell-cell signaling	8	0.0015	0.41
Locomotory behavior	5	0.0078	0.84
**Very-low-density lipoprotein particle clearance**	**2**	**0.0086**	**0.79**
Regulation of axonogenesis	3	0.012	0.81
Cell adhesion	7	0.014	0.82
**Chylomicron remnant clearance**	**2**	**0.014**	**0.77**
Biological adhesion	7	0.014	0.72
Regulation of cell cycle	5	0.015	0.69
Regulation of neuron projection development	3	0.017	0.71
**Positive regulation of cholesterol esterification**	**2**	**0.02**	**0.73**
Regulation of cell morphogenesis involved in differentiation	3	0.021	0.71
Cell projection organization	5	0.021	0.69
**Regulation of cholesterol esterification**	**2**	**0.023**	**0.69**
Regulation of cell projection organization	3	0.027	0.73
Phospholipid efflux	2	0.028	0.72
**Protein-lipid complex assembly**	**2**	**0.034**	**0.77**
**High-density lipoprotein particle remodeling**	**2**	**0.034**	**0.77**
**Plasma lipoprotein particle assembly**	**2**	**0.034**	**0.77**
**Positive regulation of steroid metabolic process**	**2**	**0.04**	**0.8**
**Negative regulation of lipid biosynthetic process**	**2**	**0.042**	**0.8**
Behavior	5	0.045	0.81
**Lipoprotein particle clearance**	**2**	**0.045**	**0.79**
Negative regulation of catalytic activity	4	0.045	0.78

Gene ontology terms related to lipoprotein metabolism indicated in bold
