# Supplementary material for: Genetic evidence for common pathways in human age-related diseases
Source: Aging Cell. 2015 Jun 15;14(5):809–17. doi: 10.1111/acel.12362 (PMC4568968; doi:10.1111/acel.12362)
Supplement: Supplementary file 10 [file acel0014-0809-sd10.pdf]

Table S7 – Comparison of SNP, Gene, and GO Term Numbers Identified by GWAS Cutoff

Disease Category	SNPs p<10-5	SNPs p<10-7	Difference (percent)	Genes p<10-5	Genes p<10-7	Difference (percent)	GO p<10-5	GO p<10-7	Difference (percent)
Cancer	455	304	33.19	377	280	25.73	1082	1036	4.25
Cardiovascular	414	207	50.00	458	256	44.10	1213	1068	11.95
Frailty	521	366	29.75	507	417	17.75	1512	1461	3.37
Metabolic	918	709	22.77	638	523	18.03	1725	1686	2.26
Neurodegenerative	424	183	56.84	371	185	50.13	1048	897	14.41

Comparison of the number of SNPs, GO terms, and genes identified in each disease category using GWAS p-value cutoff of p<10-5 versus p<10-7.
